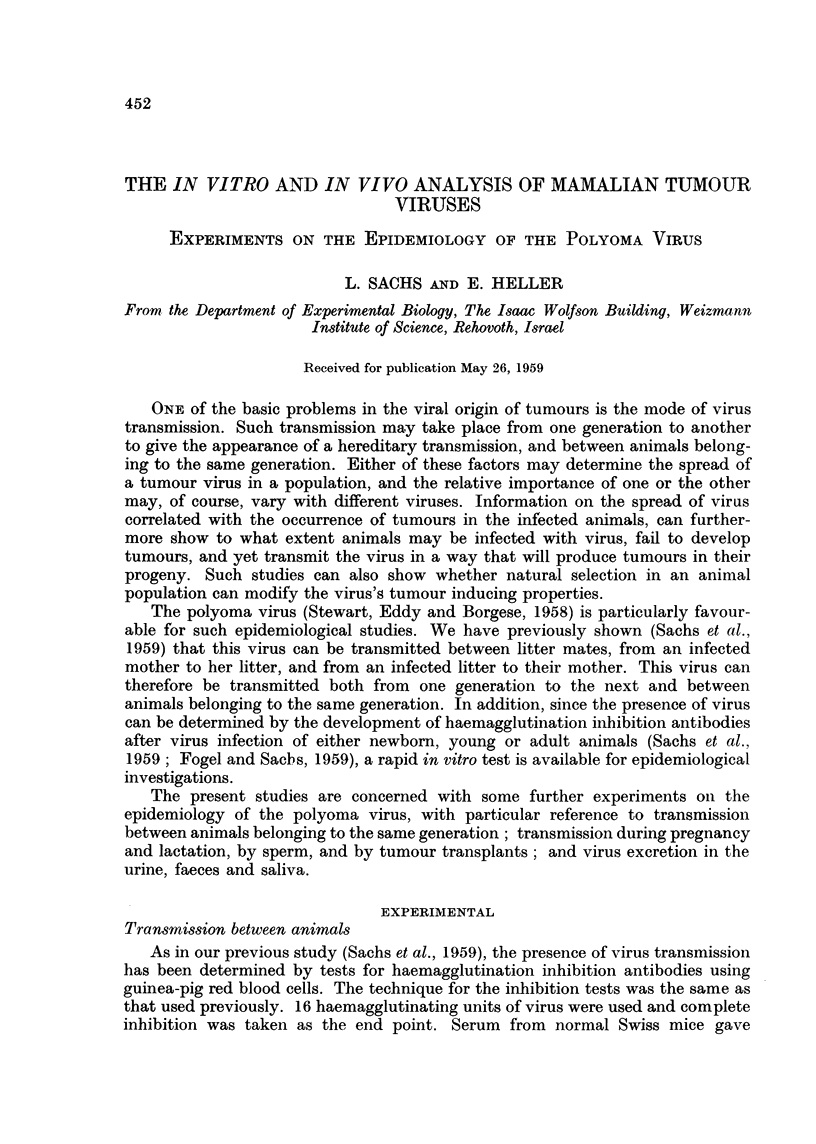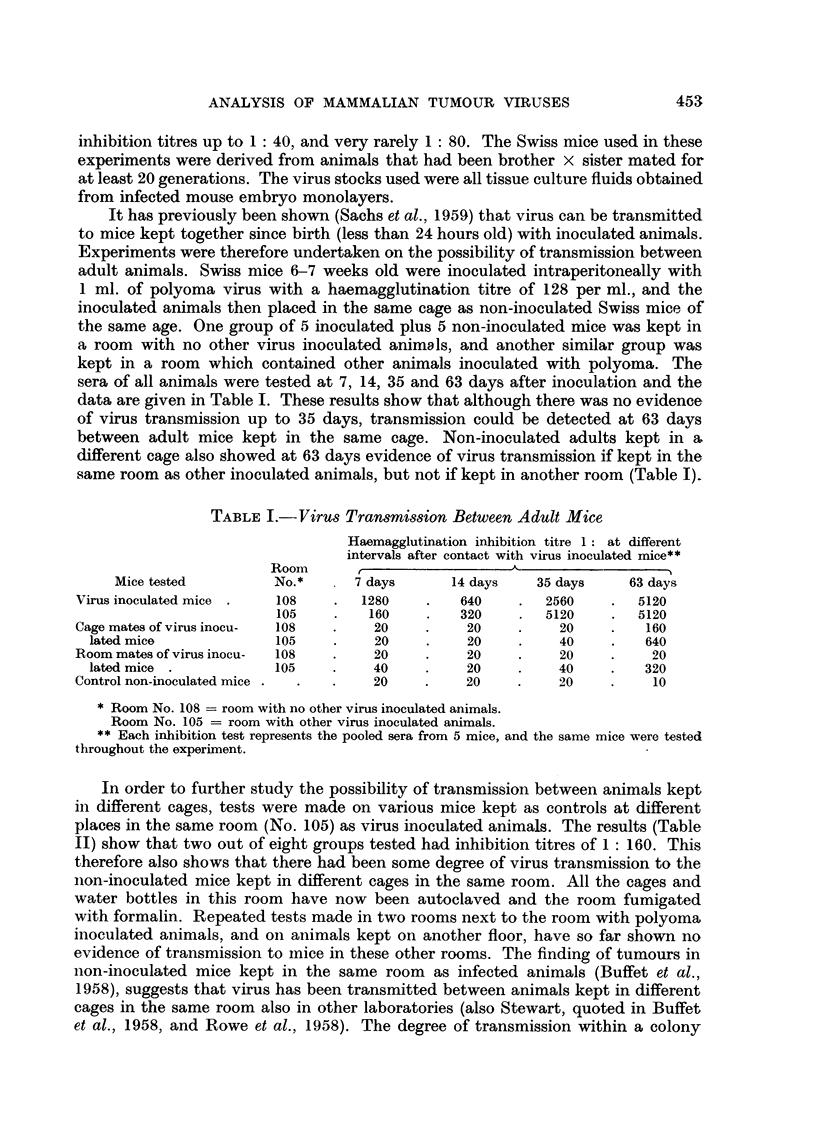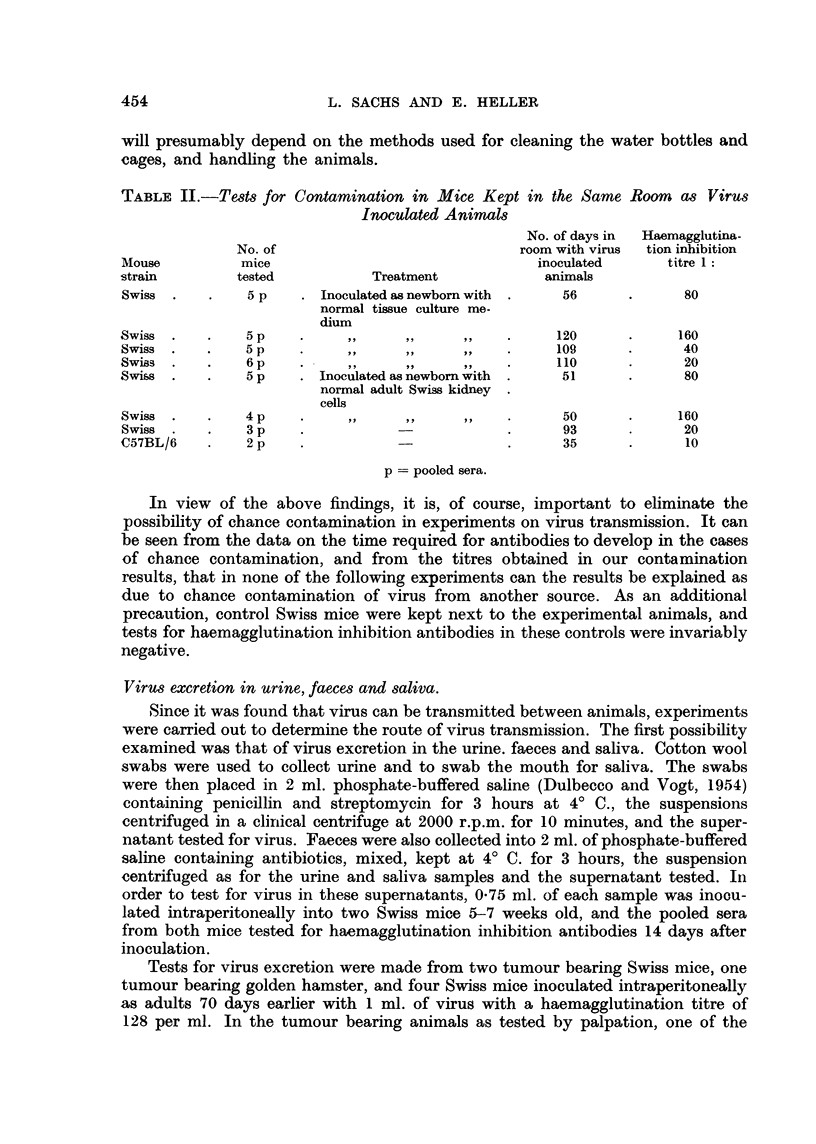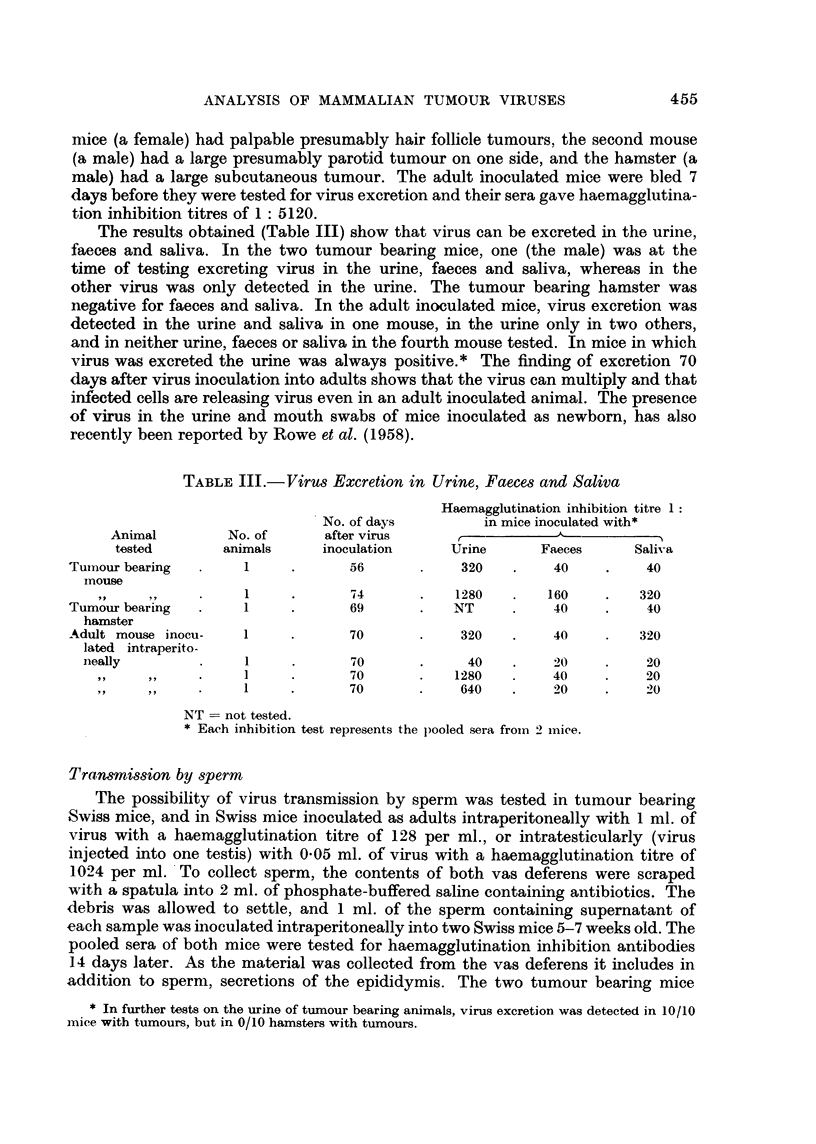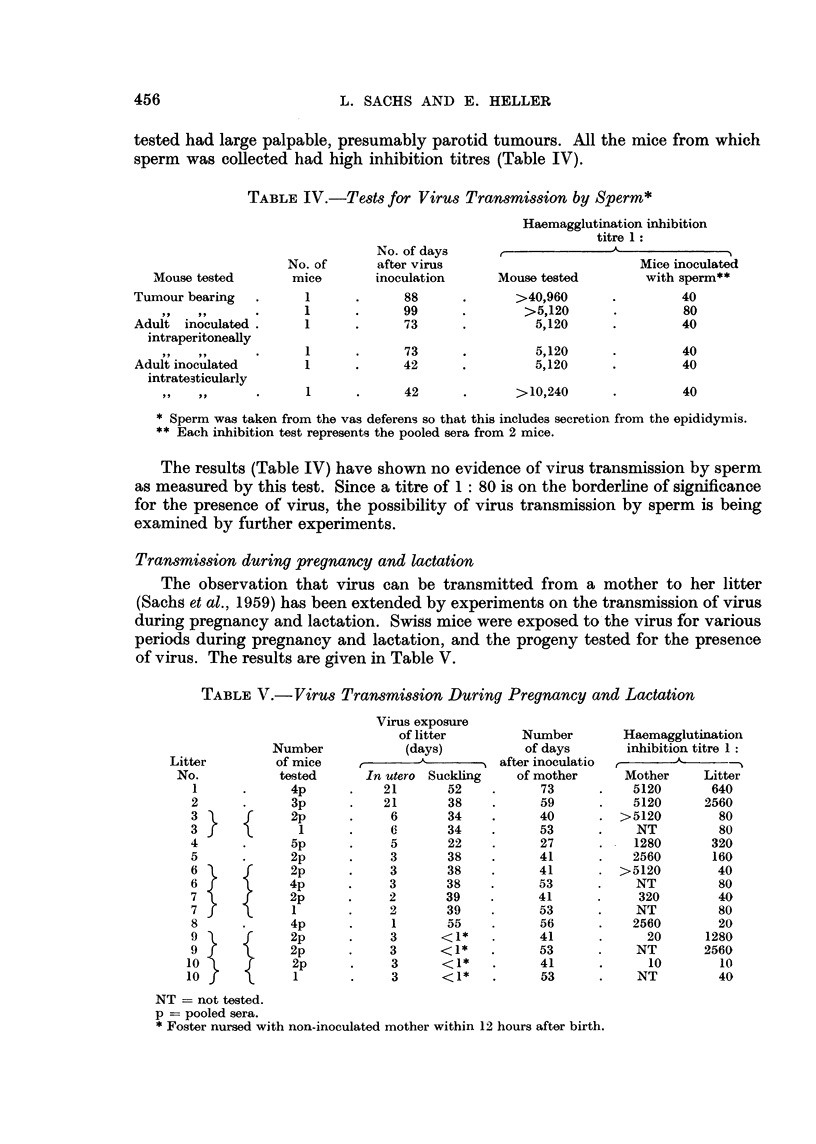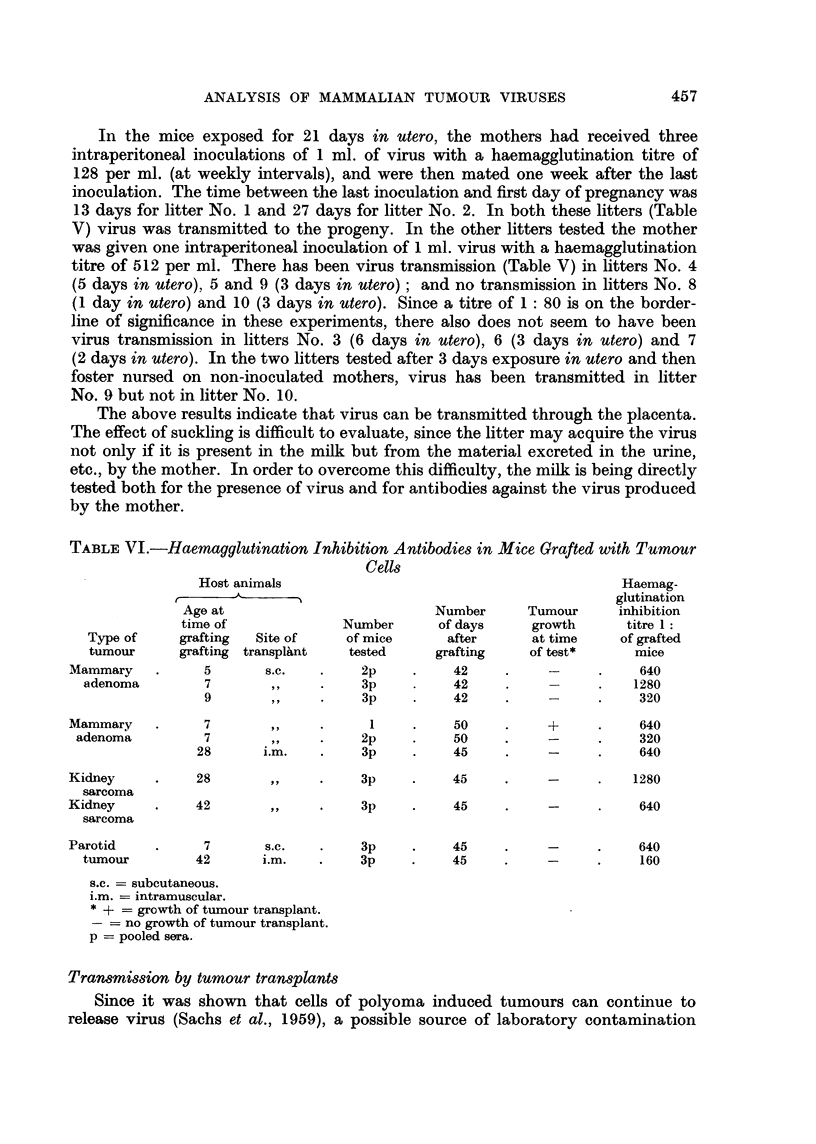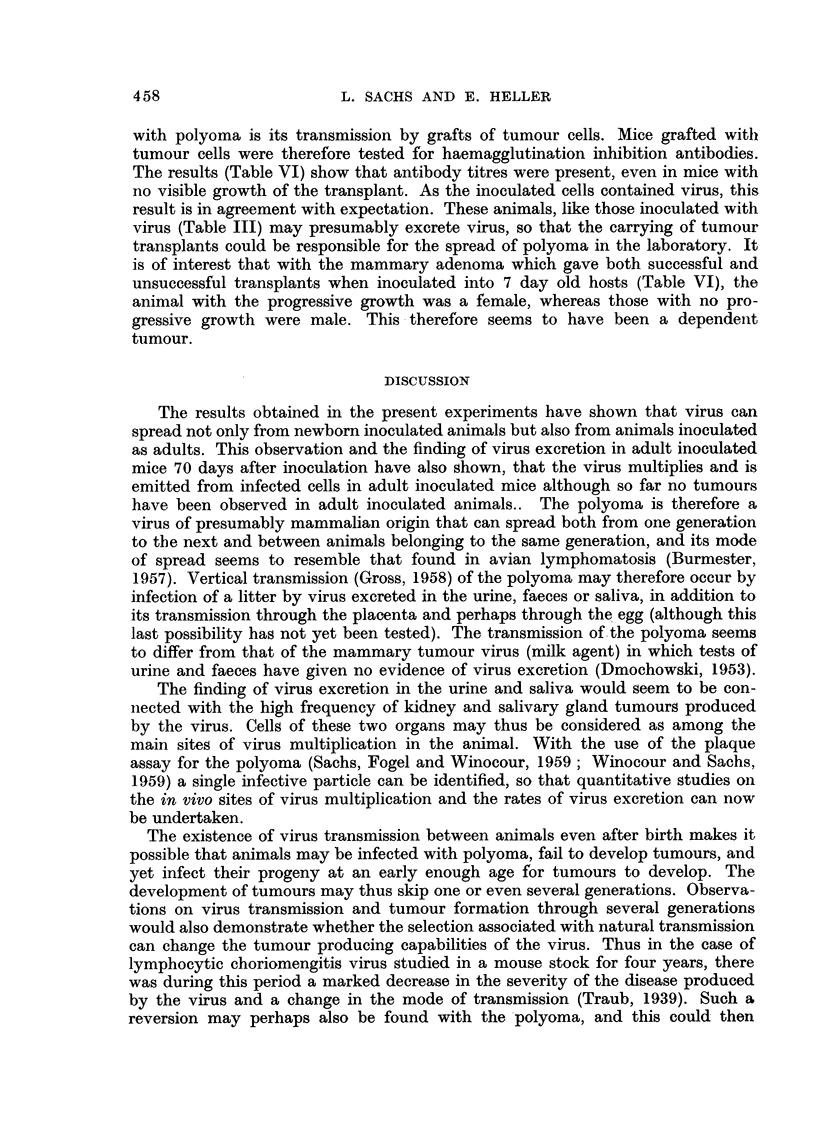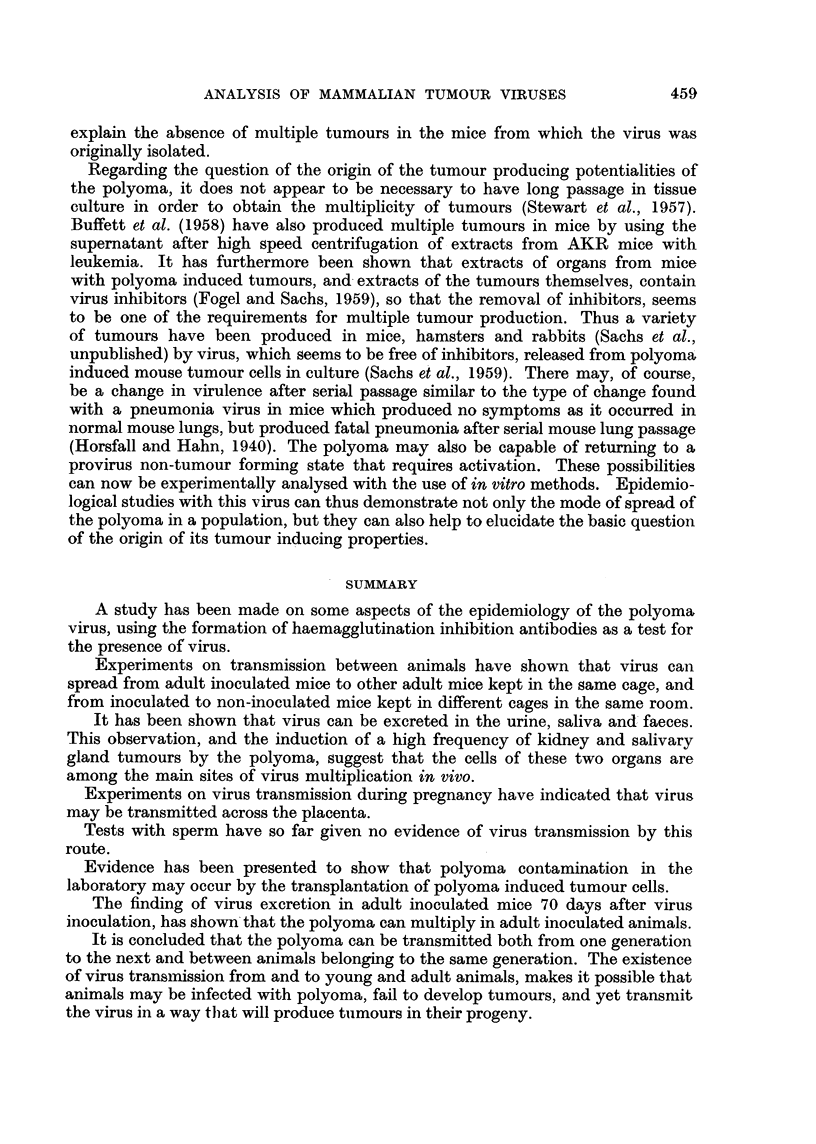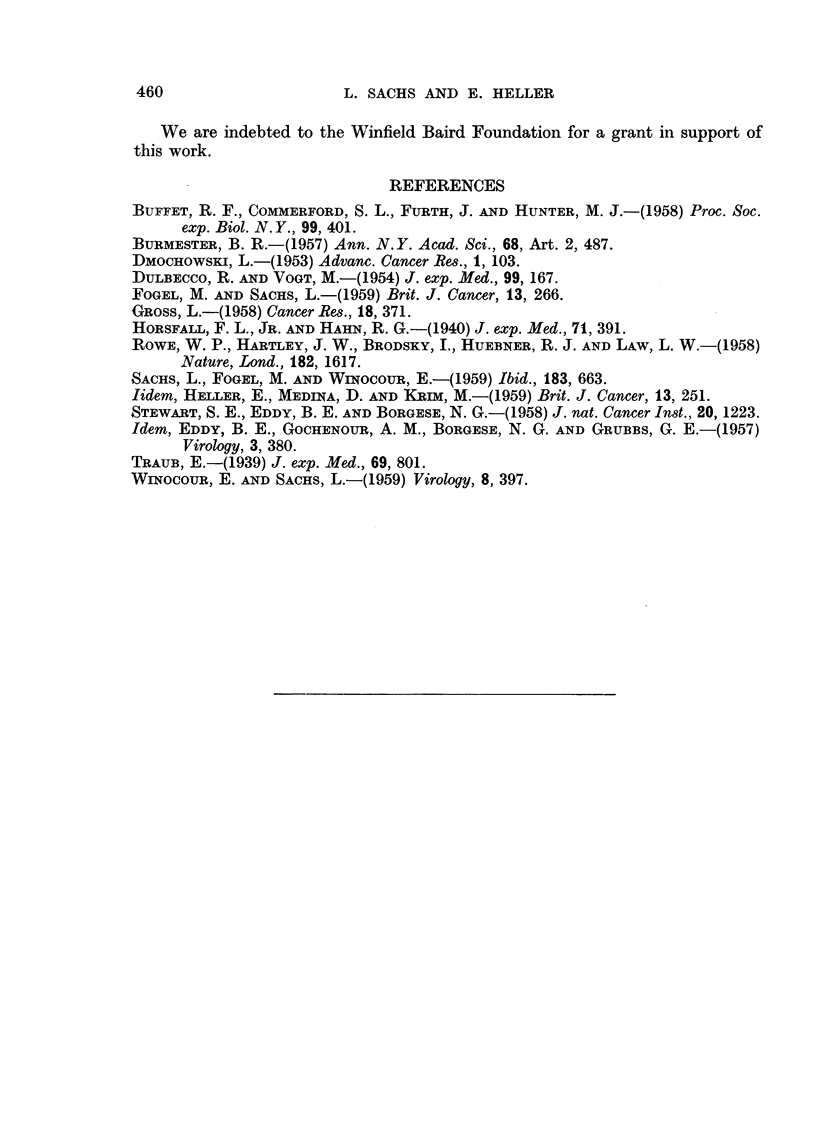# The In Vitro and In Vivo Analysis of Mammalian Tumour Viruses

**DOI:** 10.1038/bjc.1959.50

**Published:** 1959-09

**Authors:** L. Sachs, E. Heller


### THE *IN VITRO* AND *IN VIVO* ANALYSIS OF MAMALIAN TUMOUR VIRUSES

### EXPERIMENTS ON THE EPIDEMIOLOGY OF THE POLYOMA VIRUS

L. SACHS AND E. HELLER

*From the Department of Experimental Biology, The Isaac Wolfson Building, Weizmann Institute of Science, Rehovoth, Israel*

Received for publication May 26, 1959

ONE of the basic problems in the viral origin of tumours is the mode of virus transmission. Such transmission may take place from one generation to another to give the appearance of a hereditary transmission, and between animals belonging to the same generation. Either of these factors may determine the spread of a tumour virus in a population, and the relative importance of one or the other may, of course, vary with different viruses. Information on the spread of virus correlated with the occurrence of tumours in the infected animals, can furthermore show to what extent animals may be infected with virus, fail to develop tumours, and yet transmit the virus in a way that will produce tumours in their progeny. Such studies can also show whether natural selection in an animal population can modify the virus's tumour inducing properties.

The polyoma virus (Stewart, Eddy and Borgese, 1958) is particularly favourable for such epidemiological studies. We have previously shown (Sachs *et al.*, 1959) that this virus can be transmitted between litter mates, from an infected mother to her litter, and from an infected litter to their mother. This virus can therefore be transmitted both from one generation to the next and between animals belonging to the same generation. In addition, since the presence of virus can be determined by the development of haemagglutination inhibition antibodies after virus infection of either newborn, young or adult animals (Sachs *et al.*, 1959 ; Fogel and Sachs, 1959), a rapid *in vitro* test is available for epidemiological investigations.

The present studies are concerned with some further experiments on the epidemiology of the polyoma virus, with particular reference to transmission between animals belonging to the same generation ; transmission during pregnancy and lactation, by sperm, and by tumour transplants ; and virus excretion in the urine, faeces and saliva.

### EXPERIMENTAL

### *Transmission between animals*

As in our previous study (Sachs *et al.*, 1959), the presence of virus transmission has been determined by tests for haemagglutination inhibition antibodies using guinea-pig red blood cells. The technique for the inhibition tests was the same as that used previously. 16 haemagglutinating units of virus were used and complete inhibition was taken as the end point. Serum from normal Swiss mice gave inhibition titres up to 1 : 40, and very rarely 1 : 80. The Swiss mice used in these experiments were derived from animals that had been brother × sister mated for at least 20 generations. The virus stocks used were all tissue culture fluids obtained from infected mouse embryo monolayers.

It has previously been shown (Sachs *et al.*, 1959) that virus can be transmitted to mice kept together since birth (less than 24 hours old) with inoculated animals. Experiments were therefore undertaken on the possibility of transmission between adult animals. Swiss mice 6–7 weeks old were inoculated intraperitoneally with 1 ml. of polyoma virus with a haemagglutination titre of 128 per ml., and the inoculated animals then placed in the same cage as non-inoculated Swiss mice of the same age. One group of 5 inoculated plus 5 non-inoculated mice was kept in a room with no other virus inoculated animals, and another similar group was kept in a room which contained other animals inoculated with polyoma. The sera of all animals were tested at 7, 14, 35 and 63 days after inoculation and the data are given in Table I. These results show that although there was no evidence of virus transmission up to 35 days, transmission could be detected at 63 days between adult mice kept in the same cage. Non-inoculated adults kept in a different cage also showed at 63 days evidence of virus transmission if kept in the same room as other inoculated animals, but not if kept in another room (Table I).

TABLE I.—*Virus Transmission Between Adult Mice*

| Mice tested | Room No.* | Haemagglutination inhibition titre 1 : at different intervals after contact with virus inoculated mice** 7 days | 14 days | 35 days | 63 days |
|---|---|---|---|---|---|
| Virus inoculated mice | 108 | 1280 | 640 | 2560 | 5120 |
| | 105 | 160 | 320 | 5120 | 5120 |
| Cage mates of virus inoculated mice | 108 | 20 | 20 | 20 | 160 |
| | 105 | 20 | 20 | 40 | 640 |
| Room mates of virus inoculated mice | 108 | 20 | 20 | 20 | 20 |
| | 105 | 40 | 20 | 40 | 320 |
| Control non-inoculated mice | | 20 | 20 | 20 | 10 |

* Room No. 108 = room with no other virus inoculated animals.
Room No. 105 = room with other virus inoculated animals.
** Each inhibition test represents the pooled sera from 5 mice, and the same mice were tested throughout the experiment.

In order to further study the possibility of transmission between animals kept in different cages, tests were made on various mice kept as controls at different places in the same room (No. 105) as virus inoculated animals. The results (Table II) show that two out of eight groups tested had inhibition titres of 1 : 160. This therefore also shows that there had been some degree of virus transmission to the non-inoculated mice kept in different cages in the same room. All the cages and water bottles in this room have now been autoclaved and the room fumigated with formalin. Repeated tests made in two rooms next to the room with polyoma inoculated animals, and on animals kept on another floor, have so far shown no evidence of transmission to mice in these other rooms. The finding of tumours in non-inoculated mice kept in the same room as infected animals (Buffet *et al.*, 1958), suggests that virus has been transmitted between animals kept in different cages in the same room also in other laboratories (also Stewart, quoted in Buffet *et al.*, 1958, and Rowe *et al.*, 1958). The degree of transmission within a colony will presumably depend on the methods used for cleaning the water bottles and cages, and handling the animals.

TABLE II.—*Tests for Contamination in Mice Kept in the Same Room as Virus Inoculated Animals*

| Mouse strain | No. of mice tested | Treatment | No. of days in room with virus inoculated animals | Haemagglutination inhibition titre 1 : |
|---|---|---|---|---|
| Swiss | 5 p | Inoculated as newborn with normal tissue culture medium | 56 | 80 |
| Swiss | 5 p | ,, ,, ,, | 120 | 160 |
| Swiss | 5 p | ,, ,, ,, | 109 | 40 |
| Swiss | 6 p | ,, ,, ,, | 110 | 20 |
| Swiss | 5 p | Inoculated as newborn with normal adult Swiss kidney cells | 51 | 80 |
| Swiss | 4 p | ,, ,, ,, | 50 | 160 |
| Swiss | 3 p | — | 93 | 20 |
| C57BL/6 | 2 p | — | 35 | 10 |

p = pooled sera.

In view of the above findings, it is, of course, important to eliminate the possibility of chance contamination in experiments on virus transmission. It can be seen from the data on the time required for antibodies to develop in the cases of chance contamination, and from the titres obtained in our contamination results, that in none of the following experiments can the results be explained as due to chance contamination of virus from another source. As an additional precaution, control Swiss mice were kept next to the experimental animals, and tests for haemagglutination inhibition antibodies in these controls were invariably negative.

*Virus excretion in urine, faeces and saliva.*

Since it was found that virus can be transmitted between animals, experiments were carried out to determine the route of virus transmission. The first possibility examined was that of virus excretion in the urine. faeces and saliva. Cotton wool swabs were used to collect urine and to swab the mouth for saliva. The swabs were then placed in 2 ml. phosphate-buffered saline (Dulbecco and Vogt, 1954) containing penicillin and streptomycin for 3 hours at 4° C., the suspensions centrifuged in a clinical centrifuge at 2000 r.p.m. for 10 minutes, and the supernatant tested for virus. Faeces were also collected into 2 ml. of phosphate-buffered saline containing antibiotics, mixed, kept at 4° C. for 3 hours, the suspension centrifuged as for the urine and saliva samples and the supernatant tested. In order to test for virus in these supernatants, 0·75 ml. of each sample was inoculated intraperitoneally into two Swiss mice 5–7 weeks old, and the pooled sera from both mice tested for haemagglutination inhibition antibodies 14 days after inoculation.

Tests for virus excretion were made from two tumour bearing Swiss mice, one tumour bearing golden hamster, and four Swiss mice inoculated intraperitoneally as adults 70 days earlier with 1 ml. of virus with a haemagglutination titre of 128 per ml. In the tumour bearing animals as tested by palpation, one of the mice (a female) had palpable presumably hair follicle tumours, the second mouse (a male) had a large presumably parotid tumour on one side, and the hamster (a male) had a large subcutaneous tumour. The adult inoculated mice were bled 7 days before they were tested for virus excretion and their sera gave haemagglutination inhibition titres of 1 : 5120.

The results obtained (Table III) show that virus can be excreted in the urine, faeces and saliva. In the two tumour bearing mice, one (the male) was at the time of testing excreting virus in the urine, faeces and saliva, whereas in the other virus was only detected in the urine. The tumour bearing hamster was negative for faeces and saliva. In the adult inoculated mice, virus excretion was detected in the urine and saliva in one mouse, in the urine only in two others, and in neither urine, faeces or saliva in the fourth mouse tested. In mice in which virus was excreted the urine was always positive.* The finding of excretion 70 days after virus inoculation into adults shows that the virus can multiply and that infected cells are releasing virus even in an adult inoculated animal. The presence of virus in the urine and mouth swabs of mice inoculated as newborn, has also recently been reported by Rowe *et al.* (1958).

TABLE III.—*Virus Excretion in Urine, Faeces and Saliva*

| Animal tested | No. of animals | No. of days after virus inoculation | Haemagglutination inhibition titre 1 : in mice inoculated with* | | |
|---|---|---|---|---|---|
| | | | Urine | Faeces | Saliva |
| Tumour bearing mouse | 1 | 56 | 320 | 40 | 40 |
| ,, ,, | 1 | 74 | 1280 | 160 | 320 |
| Tumour bearing hamster | 1 | 69 | NT | 40 | 40 |
| Adult mouse inoculated intraperitoneally | 1 | 70 | 320 | 40 | 320 |
| | 1 | 70 | 40 | 20 | 20 |
| ,, ,, | 1 | 70 | 1280 | 40 | 20 |
| ,, ,, | 1 | 70 | 640 | 20 | 20 |

NT = not tested.
* Each inhibition test represents the pooled sera from 2 mice.

*Transmission by sperm*

The possibility of virus transmission by sperm was tested in tumour bearing Swiss mice, and in Swiss mice inoculated as adults intraperitoneally with 1 ml. of virus with a haemagglutination titre of 128 per ml., or intratesticularly (virus injected into one testis) with 0·05 ml. of virus with a haemagglutination titre of 1024 per ml. To collect sperm, the contents of both vas deferens were scraped with a spatula into 2 ml. of phosphate-buffered saline containing antibiotics. The debris was allowed to settle, and 1 ml. of the sperm containing supernatant of each sample was inoculated intraperitoneally into two Swiss mice 5–7 weeks old. The pooled sera of both mice were tested for haemagglutination inhibition antibodies 14 days later. As the material was collected from the vas deferens it includes in addition to sperm, secretions of the epididymis. The two tumour bearing mice

* In further tests on the urine of tumour bearing animals, virus excretion was detected in 10/10 mice with tumours, but in 0/10 hamsters with tumours.

tested had large palpable, presumably parotid tumours. All the mice from which sperm was collected had high inhibition titres (Table IV).

TABLE IV.—*Tests for Virus Transmission by Sperm**

| Mouse tested | No. of mice | No. of days after virus inoculation | Haemagglutination inhibition titre 1 : Mouse tested | Mice inoculated with sperm** |
|---|---|---|---|---|
| Tumour bearing | 1 | 88 | >40,960 | 40 |
| ,, ,, | 1 | 99 | >5,120 | 80 |
| Adult inoculated intraperitoneally | 1 | 73 | 5,120 | 40 |
| ,, ,, | 1 | 73 | 5,120 | 40 |
| Adult inoculated intratesticularly | 1 | 42 | 5,120 | 40 |
| ,, ,, | 1 | 42 | >10,240 | 40 |

* Sperm was taken from the vas deferens so that this includes secretion from the epididymis.
** Each inhibition test represents the pooled sera from 2 mice.

The results (Table IV) have shown no evidence of virus transmission by sperm as measured by this test. Since a titre of 1 : 80 is on the borderline of significance for the presence of virus, the possibility of virus transmission by sperm is being examined by further experiments.

*Transmission during pregnancy and lactation*

The observation that virus can be transmitted from a mother to her litter (Sachs *et al.*, 1959) has been extended by experiments on the transmission of virus during pregnancy and lactation. Swiss mice were exposed to the virus for various periods during pregnancy and lactation, and the progeny tested for the presence of virus. The results are given in Table V.

TABLE V.—*Virus Transmission During Pregnancy and Lactation*

| Litter No. | Number of mice tested | Virus exposure of litter (days) *In utero* | Suckling | Number of days after inoculatio of mother | Haemagglutination inhibition titre 1 : Mother | Litter |
|---|---|---|---|---|---|---|
| 1 | 4p | 21 | 52 | 73 | 5120 | 640 |
| 2 | 3p | 21 | 38 | 59 | 5120 | 2560 |
| 3 } | { 2p | 6 | 34 | 40 | >5120 | 80 |
| 3 } | { 1 | 6 | 34 | 53 | NT | 80 |
| 4 | 5p | 5 | 22 | 27 | 1280 | 320 |
| 5 | 2p | 3 | 38 | 41 | 2560 | 160 |
| 6 } | { 2p | 3 | 38 | 41 | >5120 | 40 |
| 6 } | { 4p | 3 | 38 | 53 | NT | 80 |
| 7 } | { 2p | 2 | 39 | 41 | 320 | 40 |
| 7 } | { 1 | 2 | 39 | 53 | NT | 80 |
| 8 | 4p | 1 | 55 | 56 | 2560 | 20 |
| 9 } | { 2p | 3 | <1* | 41 | 20 | 1280 |
| 9 } | { 2p | 3 | <1* | 53 | NT | 2560 |
| 10 } | { 2p | 3 | <1* | 41 | 10 | 10 |
| 10 } | { 1 | 3 | <1* | 53 | NT | 40 |

NT = not tested.
p = pooled sera.
* Foster nursed with non-inoculated mother within 12 hours after birth.

In the mice exposed for 21 days *in utero*, the mothers had received three intraperitoneal inoculations of 1 ml. of virus with a haemagglutination titre of 128 per ml. (at weekly intervals), and were then mated one week after the last inoculation. The time between the last inoculation and first day of pregnancy was 13 days for litter No. 1 and 27 days for litter No. 2. In both these litters (Table V) virus was transmitted to the progeny. In the other litters tested the mother was given one intraperitoneal inoculation of 1 ml. virus with a haemagglutination titre of 512 per ml. There has been virus transmission (Table V) in litters No. 4 (5 days *in utero*), 5 and 9 (3 days *in utero*) ; and no transmission in litters No. 8 (1 day *in utero*) and 10 (3 days *in utero*). Since a titre of 1 : 80 is on the borderline of significance in these experiments, there also does not seem to have been virus transmission in litters No. 3 (6 days *in utero*), 6 (3 days *in utero*) and 7 (2 days *in utero*). In the two litters tested after 3 days exposure *in utero* and then foster nursed on non-inoculated mothers, virus has been transmitted in litter No. 9 but not in litter No. 10.

The above results indicate that virus can be transmitted through the placenta. The effect of suckling is difficult to evaluate, since the litter may acquire the virus not only if it is present in the milk but from the material excreted in the urine, etc., by the mother. In order to overcome this difficulty, the milk is being directly tested both for the presence of virus and for antibodies against the virus produced by the mother.

TABLE VI.—*Haemagglutination Inhibition Antibodies in Mice Grafted with Tumour Cells*

| Type of tumour | Host animals: Age at time of grafting grafting | Host animals: Site of transplant | Number of mice tested | Number of days after grafting | Tumour growth at time of test* | Haemagglutination inhibition titre 1 : of grafted mice |
|---|---|---|---|---|---|---|
| Mammary adenoma | 5 | s.c. | 2p | 42 | — | 640 |
| | 7 | ,, | 3p | 42 | — | 1280 |
| | 9 | ,, | 3p | 42 | — | 320 |
| Mammary adenoma | 7 | ,, | 1 | 50 | + | 640 |
| | 7 | ,, | 2p | 50 | — | 320 |
| | 28 | i.m. | 3p | 45 | — | 640 |
| Kidney sarcoma | 28 | ,, | 3p | 45 | — | 1280 |
| Kidney sarcoma | 42 | ,, | 3p | 45 | — | 640 |
| Parotid tumour | 7 | s.c. | 3p | 45 | — | 640 |
| | 42 | i.m. | 3p | 45 | — | 160 |

s.c. = subcutaneous.
i.m. = intramuscular.
* + = growth of tumour transplant.
— = no growth of tumour transplant.
p = pooled sera.

*Transmission by tumour transplants*

Since it was shown that cells of polyoma induced tumours can continue to release virus (Sachs *et al.*, 1959), a possible source of laboratory contamination with polyoma is its transmission by grafts of tumour cells. Mice grafted with tumour cells were therefore tested for haemagglutination inhibition antibodies. The results (Table VI) show that antibody titres were present, even in mice with no visible growth of the transplant. As the inoculated cells contained virus, this result is in agreement with expectation. These animals, like those inoculated with virus (Table III) may presumably excrete virus, so that the carrying of tumour transplants could be responsible for the spread of polyoma in the laboratory. It is of interest that with the mammary adenoma which gave both successful and unsuccessful transplants when inoculated into 7 day old hosts (Table VI), the animal with the progressive growth was a female, whereas those with no progressive growth were male. This therefore seems to have been a dependent tumour.

### DISCUSSION

The results obtained in the present experiments have shown that virus can spread not only from newborn inoculated animals but also from animals inoculated as adults. This observation and the finding of virus excretion in adult inoculated mice 70 days after inoculation have also shown, that the virus multiplies and is emitted from infected cells in adult inoculated mice although so far no tumours have been observed in adult inoculated animals.. The polyoma is therefore a virus of presumably mammalian origin that can spread both from one generation to the next and between animals belonging to the same generation, and its mode of spread seems to resemble that found in avian lymphomatosis (Burmester, 1957). Vertical transmission (Gross, 1958) of the polyoma may therefore occur by infection of a litter by virus excreted in the urine, faeces or saliva, in addition to its transmission through the placenta and perhaps through the egg (although this last possibility has not yet been tested). The transmission of the polyoma seems to differ from that of the mammary tumour virus (milk agent) in which tests of urine and faeces have given no evidence of virus excretion (Dmochowski, 1953).

The finding of virus excretion in the urine and saliva would seem to be connected with the high frequency of kidney and salivary gland tumours produced by the virus. Cells of these two organs may thus be considered as among the main sites of virus multiplication in the animal. With the use of the plaque assay for the polyoma (Sachs, Fogel and Winocour, 1959 ; Winocour and Sachs, 1959) a single infective particle can be identified, so that quantitative studies on the *in vivo* sites of virus multiplication and the rates of virus excretion can now be undertaken.

The existence of virus transmission between animals even after birth makes it possible that animals may be infected with polyoma, fail to develop tumours, and yet infect their progeny at an early enough age for tumours to develop. The development of tumours may thus skip one or even several generations. Observations on virus transmission and tumour formation through several generations would also demonstrate whether the selection associated with natural transmission can change the tumour producing capabilities of the virus. Thus in the case of lymphocytic choriomengitis virus studied in a mouse stock for four years, there was during this period a marked decrease in the severity of the disease produced by the virus and a change in the mode of transmission (Traub, 1939). Such a reversion may perhaps also be found with the polyoma, and this could then explain the absence of multiple tumours in the mice from which the virus was originally isolated.

Regarding the question of the origin of the tumour producing potentialities of the polyoma, it does not appear to be necessary to have long passage in tissue culture in order to obtain the multiplicity of tumours (Stewart *et al.*, 1957). Buffett *et al.* (1958) have also produced multiple tumours in mice by using the supernatant after high speed centrifugation of extracts from AKR mice with leukemia. It has furthermore been shown that extracts of organs from mice with polyoma induced tumours, and extracts of the tumours themselves, contain virus inhibitors (Fogel and Sachs, 1959), so that the removal of inhibitors, seems to be one of the requirements for multiple tumour production. Thus a variety of tumours have been produced in mice, hamsters and rabbits (Sachs *et al.*, unpublished) by virus, which seems to be free of inhibitors, released from polyoma induced mouse tumour cells in culture (Sachs *et al.*, 1959). There may, of course, be a change in virulence after serial passage similar to the type of change found with a pneumonia virus in mice which produced no symptoms as it occurred in normal mouse lungs, but produced fatal pneumonia after serial mouse lung passage (Horsfall and Hahn, 1940). The polyoma may also be capable of returning to a provirus non-tumour forming state that requires activation. These possibilities can now be experimentally analysed with the use of *in vitro* methods. Epidemiological studies with this virus can thus demonstrate not only the mode of spread of the polyoma in a population, but they can also help to elucidate the basic question of the origin of its tumour inducing properties.

### SUMMARY

A study has been made on some aspects of the epidemiology of the polyoma virus, using the formation of haemagglutination inhibition antibodies as a test for the presence of virus.

Experiments on transmission between animals have shown that virus can spread from adult inoculated mice to other adult mice kept in the same cage, and from inoculated to non-inoculated mice kept in different cages in the same room.

It has been shown that virus can be excreted in the urine, saliva and faeces. This observation, and the induction of a high frequency of kidney and salivary gland tumours by the polyoma, suggest that the cells of these two organs are among the main sites of virus multiplication *in vivo*.

Experiments on virus transmission during pregnancy have indicated that virus may be transmitted across the placenta.

Tests with sperm have so far given no evidence of virus transmission by this route.

Evidence has been presented to show that polyoma contamination in the laboratory may occur by the transplantation of polyoma induced tumour cells.

The finding of virus excretion in adult inoculated mice 70 days after virus inoculation, has shown that the polyoma can multiply in adult inoculated animals.

It is concluded that the polyoma can be transmitted both from one generation to the next and between animals belonging to the same generation. The existence of virus transmission from and to young and adult animals, makes it possible that animals may be infected with polyoma, fail to develop tumours, and yet transmit the virus in a way that will produce tumours in their progeny.

We are indebted to the Winfield Baird Foundation for a grant in support of this work.

### REFERENCES

BUFFET, R. F., COMMERFORD, S. L., FURTH, J. AND HUNTER, M. J.—(1958) *Proc. Soc. exp. Biol. N.Y.*, **99**, 401.
BURMESTER, B. R.—(1957) *Ann. N.Y. Acad. Sci.*, **68**, Art. 2, 487.
DMOCHOWSKI, L.—(1953) *Advanc. Cancer Res.*, **1**, 103.
DULBECCO, R. AND VOGT, M.—(1954) *J. exp. Med.*, **99**, 167.
FOGEL, M. AND SACHS, L.—(1959) *Brit. J. Cancer*, **13**, 266.
GROSS, L.—(1958) *Cancer Res.*, **18**, 371.
HORSFALL, F. L., JR. AND HAHN, R. G.—(1940) *J. exp. Med.*, **71**, 391.
ROWE, W. P., HARTLEY, J. W., BRODSKY, I., HUEBNER, R. J. AND LAW, L. W.—(1958) *Nature, Lond.*, **182**, 1617.
SACHS, L., FOGEL, M. AND WINOCOUR, E.—(1959) *Ibid.*, **183**, 663.
*Iidem*, HELLER, E., MEDINA, D. AND KRIM, M.—(1959) *Brit. J. Cancer*, **13**, 251.
STEWART, S. E., EDDY, B. E. AND BORGESE, N. G.—(1958) *J. nat. Cancer Inst.*, **20**, 1223.
*Idem*, EDDY, B. E., GOCHENOUR, A. M., BORGESE, N. G. AND GRUBBS, G. E.—(1957) *Virology*, **3**, 380.
TRAUB, E.—(1939) *J. exp. Med.*, **69**, 801.
WINOCOUR, E. AND SACHS, L.—(1959) *Virology*, **8**, 397.